# An integrative pan-cancer analysis reveals the oncogenic role of mutS homolog 6 (MSH6) in human tumors

**DOI:** 10.18632/aging.203745

**Published:** 2021-12-07

**Authors:** Haibo Zhan, Fengbo Mo, Qiang Xu, Song Wang, Bin Zhang, Xuqiang Liu, Min Dai, Hucheng Liu

**Affiliations:** 1 Department of Orthopedics, The First Affiliated Hospital of Nanchang University, Nanchang 330006, Jiangxi, China; 2 Artificial Joints Engineering and Technology Research Center of Jiangxi Province, Nanchang 330006, Jiangxi, China

**Keywords:** MSH6, pan-cancer, prognosis, phosphorylation, immune infiltration

## Abstract

There are three most important mismatch repair genes in the mismatch repair system, MSH6 is one of them and it plays an essential role in DNA mismatch repair. Several emerging cell- or animal-based studies have verified that MSH6 mutations are closely linked to the occurrence, progression or metastasis of cancer, but there is still no practicable pan-cancer analysis. On account of the available datasets of the cancer genome atlas (TCGA) and Gene expression omnibus (GEO), a comprehensive analysis of the potential carcinogenic effects of the MSH6 gene was conducted in 33 human cancers. MSH6 was highly expressed in most cancers, and the high expression of MSH6 was associated with poor overall survival prognosis of patients with multiple cancers, such as adrenocortical carcinoma. MSH6 mutations occurred in most cancers and were closely related to the prognosis of cancer patients. Increased phosphorylation levels of S227 and S830 were noted in several tumors, including breast cancer and colon cancer. MSH6 expression was also observed to be correlated with cancer-associated fibroblasts and CD8^+^ T-cells infiltration levels in various cancer types, e. g. pancreatic adenocarcinoma or testicular germ cell tumors. Furthermore, pathway enrichment analysis demonstrated that the main biological activities of MSH6 were related to ATPase activity, mismatch repair, and DNA metabolism-related functions. Altogether, our pan-cancer research has suggested that the MSH6 expression level was closely related to the carcinogenesis and prognosis of certain tumors, which helps to know the effect of MSH6 in tumorigenesis from the point of view of clinical tumor samples.

## INTRODUCTION

Mismatch repair genes are DNA damage response pathway’s prominent components, which is responsible for maintaining genome integrity [1], including MLH1 (mutL homolog 1), MSH2 (mutS homolog 2), PMS2 (postmeiotic segregation increased 2), MSH6 (mutS homolog 6), etc. The human MSH6 protein, also known as GTBP or p160, is one of the three most important mismatch repair proteins in the post-replicative DNA mismatch repair system (MMR)’s MutS family, which exists in mammalian cells, primarily on the short arm of chromosome 2, and plays a core role in repairing mismatched DNA bases [2, 3]. Common to all MutS homologues, MSH6 contains a Walker-A/B adenine nucleotide motif of approximately 150 amino acids, which is a highly conserved sequence with intrinsic ATPase activity [4]. In the process of DNA mismatch binding dissociation, the encoded protein can heterodimerize with MSH2 to form mismatch recognition complex, and exchange ADP and ATP as bidirectional molecular switch [5, 6]. The human MSH6 protein can be split into five conserved domains (MutS_1~5) comparable to *E. coli* MutS, and the disordered N-terminal PWWP domain [7, 8]. Within these five domains, the specific biochemical functions of MSH6 have been driven based on the sequence differences in MSH2 [9]. Previous studies have shown that abnormal expression of the MSH6 gene and its transcription characteristics have been detected in many cancer types [10–13].

The available GEO database and publicly funded TCGA project contain functional genomics datasets of human different tumors, aiming at cataloguing and discovering major carcinogenic genome alterations to create the cancer genome profiles’ comprehensive “atlas” [14–16]. Research on individual cancer types and comprehensive pan-cancer analysis have provided new insights into the occurrence and development of tumors. In recent years, the close relationship between MSH6 and tumor has also been continuously discovered. Our research team has been committed to the study of this MSH6 protein with different functions and reported the functional connection between MSH6 and the tumorigenesis and development of osteosarcoma [17]. However, currently, no pan-cancer analysis has been performed to comprehensively evaluate the relationship between MSH6 expression and the carcinogenesis and clinical prognosis of a variety of tumor types.

Through this paper, we will reveal the oncogenic role of human MSH6 (NM_000179 for mRNA or NP_000170.1 for protein) in human tumors. TCGA project and GEO database were used to perform pan-cancer analysis of MSH6 for the first time, and systematically described the expression differences, prognostic value, protein phosphorylation as well as relevant cellular pathways of MSH6 in different cancer types. The genetic alteration status and prognostic value of MSH6 across multiple cancer types and the relationship with immune cell infiltration were also investigated. Taken together, our research provided a new understanding of the potential effect of MSH6 in the pathogenesis or in clinical prognosis of various different cancers.

## RESULTS

### MSH6 expression in pan-cancer

**Figure 1:**
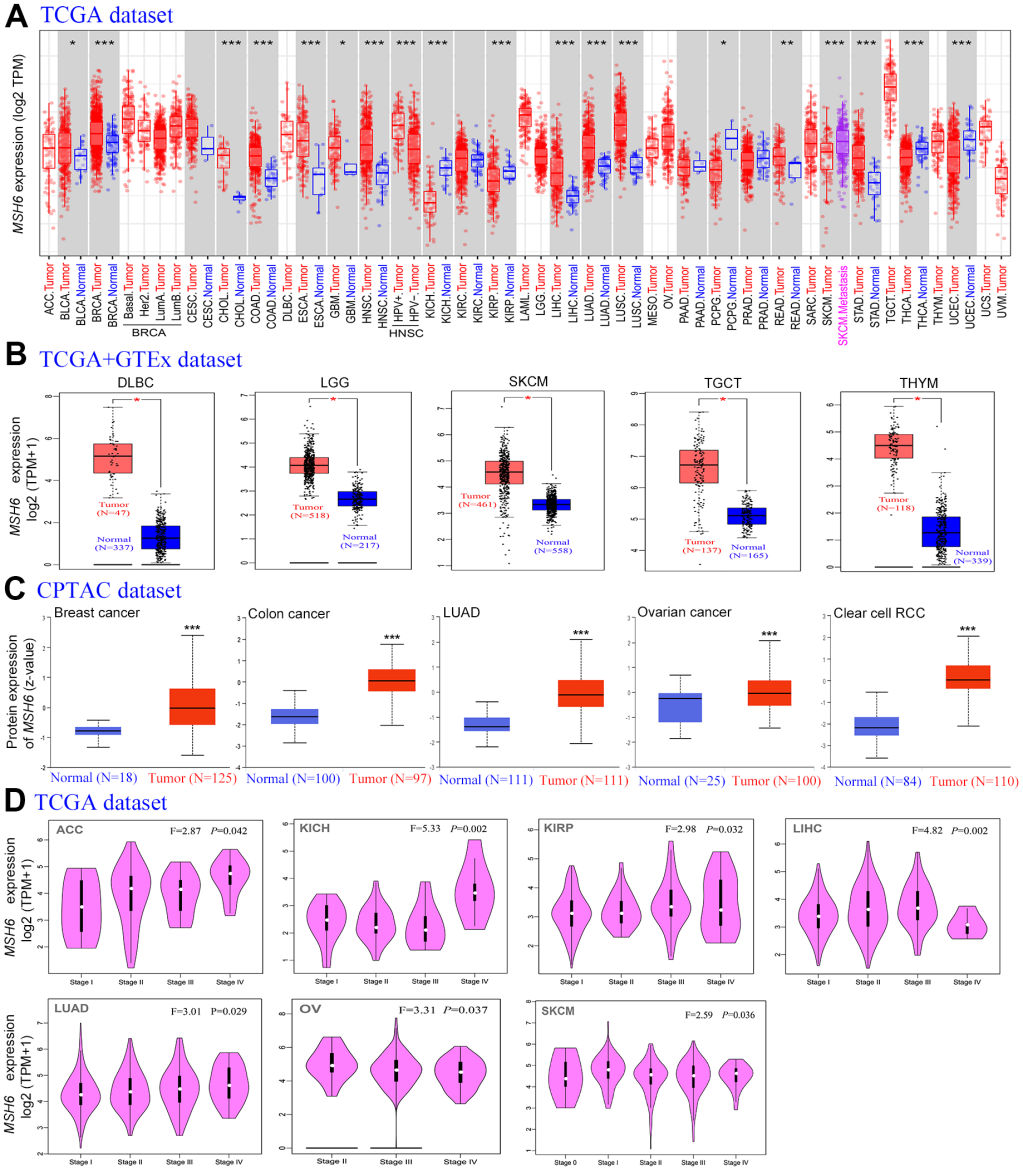
**The MSH6 expression level in different tumor tissues and stages.** (**A**) The TCGA project’s MSH6 gene expression difference in different tumors or specific tumor subtype tissues and adjacent normal tissues was analyzed by TIMER2. *P<0.05; **P<0.01; ***P<0.001. (**B**) In the GTEx database, the corresponding normal tissues were applied as controls, and GEPIA2 was applied to analyze the expression status of MSH6 gene in LGG, SKCM, DLBC, TGCT and THYM tumors. *P <0.05. (**C**) Difference of the MSH6 total protein expression between normal and tumor tissues of breast cancer, lung adenocarcinoma, colon cancer, ovarian cancer and clear cell RCC were analyzed based on the CPTAC dataset. ***P<0.001. (**D**) On the basis of the TCGA dataset, GEPIA2 was applied to analyze the expression level of MSH6 gene by the different pathological stages (stage I, II, III, IV and V) in LUAD, KICH, LIHC, ACC, KIRP, OV and SKCM tumors.

We utilized the TIMER2 tool firstly to analyze MSH6’s expression status in the TCGA project’s different cancer types. In Figure 1A, MSH6’sexpression level in the tumor tissues of HNSC [HPV (Human papillomavirus) +/-] (Head and neck squamous cell carcinoma), LUSC (Lung squamous cell carcinoma), COAD (Colon adenocarcinoma), CHOL (Cholangiocarcinoma), BRCA (Breast invasive carcinoma), ESCA (Esophageal carcinoma), BLCA (Bladder urothelial carcinoma), LIHC (Liver hepatocellular carcinoma), LUAD (Lung adenocarcinoma), STAD (Stomach adenocarcinoma) (P<0.001), READ (Rectum adenocarcinoma) (P<0.01) and GBM (Glioblastoma multiforme (P<0.05) is all higher than that of adjacent normal tissues. But the MSH6 expression level in the tumor tissues of KIRP (Kidney renal papillary cell carcinoma), KICH (renal hepatocellular carcinoma), UCEC (Uterine corpus endometrial carcinoma) (P<0.001), THCA (Thyroid carcinoma) and PCPG (Pheochromocytoma and paraganglioma) (P<0.05) is lower than that in adjacent normal tissues.

Moreover, as the TCGA project lacks information on the corresponding normal tissues of certain tumors, the TCGA and GTEx databases for analysis were combined. In Figure 1B, the expression level of MSH6 in the tumor tissues of LGG (Brain lower grade glioma), DLBC (Lymphoid neoplasm diffuse large B-cell lymphoma), TGCT (Testicular Germ Cell Tumors) SKCM (Skin cutaneous melanoma) and THYM (Thymoma) (P<0.05) is all higher than the corresponding normal tissues compared with the normal tissues of the corresponding tumors in the GTEx dataset. However, in the expression level of MSH6 between tumor and adjacent normal tissues in other tumors, we did not obtain significant differences, including LAML (Acute myeloid leukemia), ACC (Adrenocortical carcinoma) or OV (Ovarian serous cystadenocarcinoma) (Supplementary Figure 1A).

In order to make clear the protein expression level of MSH6 in different tumors, protein expression analysis on the CPTAC dataset was performed. As shown in Figure 1C, the MSH6 total protein expression level in the primary tumor tissues of colon cancer, breast cancer, ovarian cancer, LUAD, and clear cell RCC was all higher than that of normal tissues (all P<0.001).

In addition, the “Pathological Stage Plot” module of GEPIA2 was also used to analyze the relationship between MSH6 expression levels and different tumor pathological stages. In Figure 1D, MSH6 expression levels are significantly different in various pathological stages of tumors such as ACC, KIRP, LUAD, LIHC, KICH, OV and SKCM (P<0.05) but not others (Supplementary Figure 1B–1E).

### In pan-cancer, the expression of MSH6 is associated with prognosis

**Figure 2:**
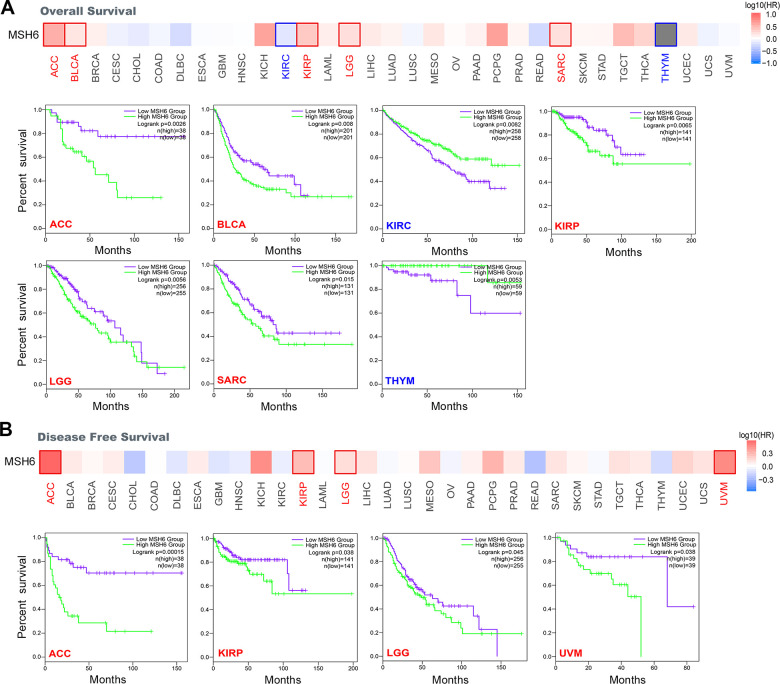
**Correlation between MSH6 gene expression and survival prognosis of all TCGA tumors were analyzed by using the GEPIA2 tool.** (**A**) Overall survival analysis. (**B**) Disease-free survival analysis. The positive results with significant differences were given through survival map and Kaplan-Meier curves.

In cancer, according to the expression level of MSH6, we divided cancer cases into two groups of MSH6 high-expression and MSH6 low-expression. Subsequently, we applied TCGA and GEO datasets to find the correlation between MSH6 expression and the prognosis of different cancer patients. In Figure 2A, we found that highly expressed MSH6 was linked with poor prognosis of OS (Overall survival) for cancer patients with ACC (P=0.0026), as well as KIRP (P=0.065), BLCA (P=0.008), SARC (Sarcoma) (P=0.015) and LGG (P=0.0056). However, the MSH6 gene low expression was linked with worse OS prognosis of KIRC (P=0.0082) and THYM (P=0.0053). In addition, DFS (Disease-free survival) analysis showed that high expression of MSH6 was correlated to poor prognosis for cancers of KIRP (P=0.038), ACC (P=0.00015), UVM (Uveal melanoma) (P=0.038) and LGG (P=0.045) (Figure 2B).

Moreover, Kaplan-Meier plotter tool was also applied to analyze the survival and prognosis data of different cancer patients. In Supplementary Figure 2A, MSH6’s high expression was linked with poor PFS (Progression-free survival) (P=0.0034), OS (P=0.022) and PPS (Post-progression survival) (P=0.048) in patients with ovarian cancer. In addition, MSH6’s high expression level was also significantly linked to poor FP (First progression) (P=2.3e-09) and OS (P=2.8e-09) in lung cancer patients (Supplementary Figure 2B). By contrast, MSH6’s low expression level was significantly linked with poor FP (P=0.019), OS (P=6e-04) and PPS (P=1.7e-05) prognosis in patients with gastric cancer (Supplementary Figure 2C). Meanwhile, MSH6’s high expression was also linked with poor PFS (P=0.0036), OS (P=0.012) and RFS (Relapse-free survival) (P=0.0034) prognosis of liver cancer patients (Supplementary Figure 2D). In addition, we also detected a relationship between the high expression level of MSH6 and the poor OS (P=0.00052), PFS (P=0.007), RFS (P=6.5e-14) and DMFS (Distant metastasis-free survival) (P=0.00022) prognosis of breast cancer patients (Supplementary Figure 2E). The above results indicate that the MSH6’s expression level is correlated to the prognosis of pan-cancer patients, but different cancer patients are also different.

### The genetic alteration of MSH6 in pan-cancer

**Figure 3:**
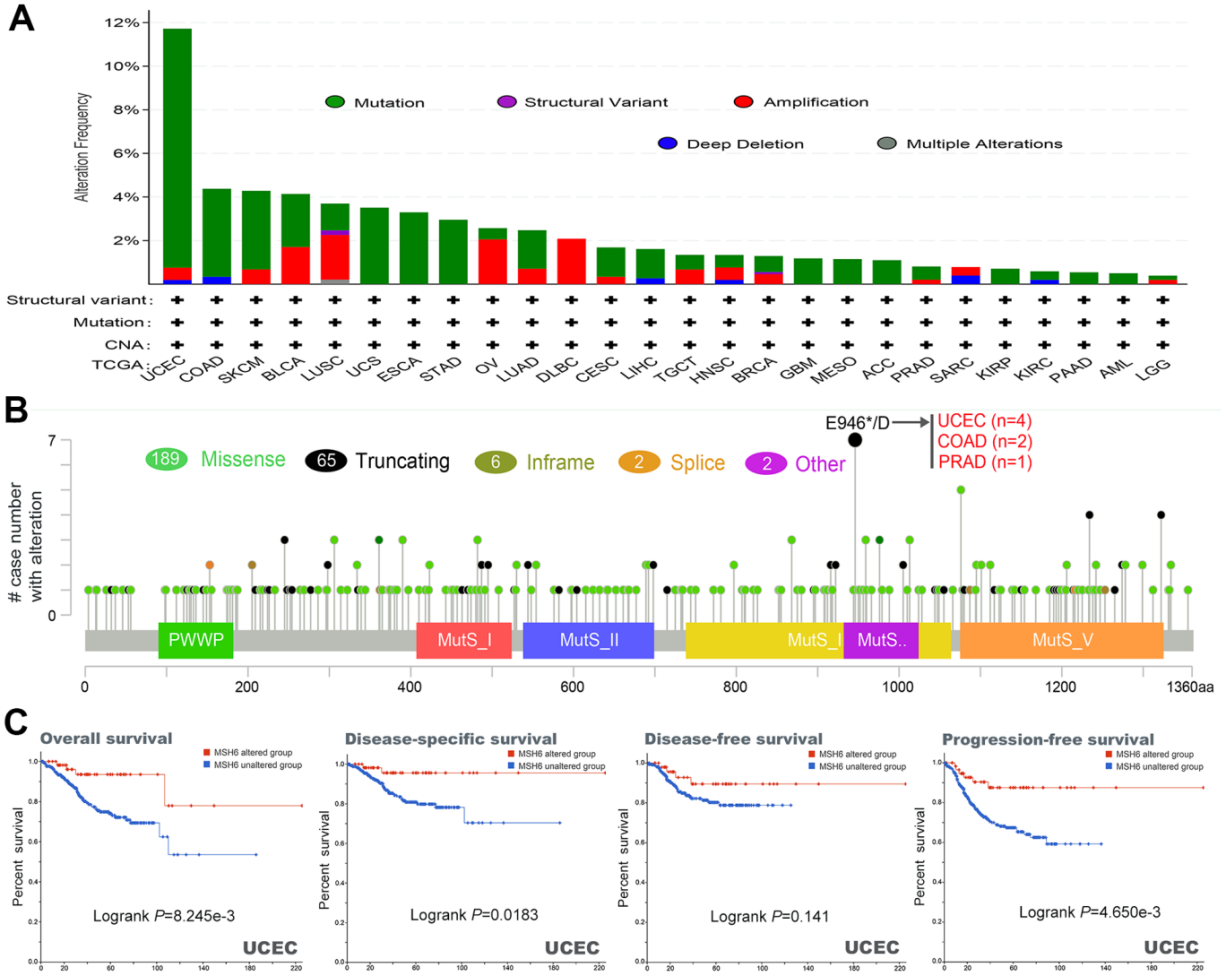
**Mutation characteristics and prognostic value of MSH6 gene in different kind of tumors of TCGA were analyzed by using the cBioPortal tool.** (**A**) These are the mutation type and alteration frequency in various tumors. (**B**) The mutation site of MSH6. (**C**) The potential correlation between MSH6 mutation status and overall, disease-free, disease-specific and progression-free survival prognoses of UCEC.

In different tumor samples of TCGA project, the MSH6 genetic alteration status was gained from cBioPortal. In Figure 3A, the MSH6 gene has the highest alteration frequency (>10%) with “mutation” as the primary type in patients with uterine tumors. In addition, CAN’s “amplification” type was the primary type of genetic alteration in the LUSC patients, with an alteration frequency of approximately 2% (Figure 3A). What is noteworthy is that all DLBC patients with genetic alterations (~2% frequency) had MSH6’s gene copy number amplification (Figure 3A). In Figure 3B, the types, location and number of cases of MSH6 genetic alteration can be further shown. It is found that missense mutations were the primary type of MSH6 gene mutation, and E946*/D alterations in the MutS_IV domain were detected in 2 cases of COAD, 4 cases of UCEC and 1 case of PRAD patients (Figure 3B). It can induce a frameshift mutation of the MSH6 gene, which translates from E (Glutamic) to D (Aspartic) at position 946 of the MSH6 protein, and the subsequent MSH6 protein truncation. Then, the potential relationship between genetic alteration of MSH6 and the clinical survival prognosis of patients with different types of cancer were also further analyzed. In Figure 3C, UCEC cancer patients with altered MSH6 indicated better prognosis in DSS (Disease-specific survival) (P=0.0183), OS (P=8.245e-03) and PFS (P=4.650e-03), but not in DFS (Disease-free survival) (P=0.141), compared with patients without MSH6 alteration. These results indicate that the MSH6’s expression status in pan-cancer is associated with MSH6 amplification and copy number gain, and the genetic alteration of MSH6 is closely linked to the various cancer patients’ clinical survival prognosis.

### Difference of MSH6 protein phosphorylation level in pan-cancer

**Figure 4:**
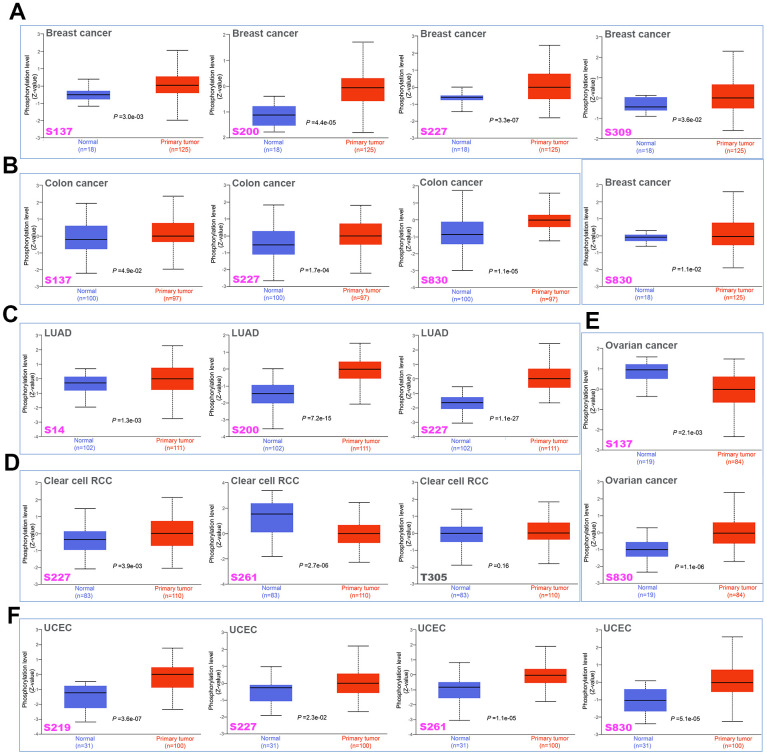
**Phosphorylation differences of MSH6 protein in various cancers of TCGA.** Based on the CPTAC dataset, the expression differences of MSH6 phosphoprotein (NP_000170.1, S14, S137, S200, S219, S227, S261, S309, and S830 sites) between normal tissue and tumor tissue from selected tumors were detected through the UALCAN. (**A**) Breast cancer. (**B**) Colon cancer. (**C**) LUAD. (**D**) Clear cell RCC. (**E**) Ovarian cancer. (**F**) UCEC.

The CPTAC dataset was applied to analyze the differences in the phosphorylation levels of MSH6 in normal and primary tumor tissues of six different tumors (colon cancer, breast cancer, LUAD, ovarian cancer, clear cell RCC and UCEC). S227 locus of MSH6 demonstrates higher phosphorylation level in primary tumor tissues of colon cancer, LUAD, breast cancer, clear cell RCC and UCEC compared with those normal tissues (Figure 4A–4D, 4F, all P <0.05). Similarly, the S830 locus within the MutS_III domain of MSH6 also indicates a higher phosphorylation level in primary tumor tissues of breast cancer, ovarian cancer colon cancer and UCEC in comparison with normal tissues (Figure 4A, 4B, 4E, 4F, all P<0.05). In contrast, in comparison with normal tissues, the S261 locus and S137 locus of MSH6 demonstrated a lower phosphorylation level of primary tumor tissues of clear cell RCC and ovarian cancer, respectively (Figure 4D, 4E, all P <0.05). Subsequently, the PhosphoNET database was also applied to further analyze the phosphorylation sites of MSH6 identified from the CPTAC dataset, and found that the phosphorylation of MSH6 in S227, S261, S830 [18] and S137 [19] in the cell cycle was confirmed by the previous publications experimentally. However, these phosphorylation sites deserve further molecular testing to further find the potential effect of these phosphorylation sites in the initiation and progression of different tumors.

### MSH6 is associated with tumor immune infiltration in pan-cancer

**Figure 5:**
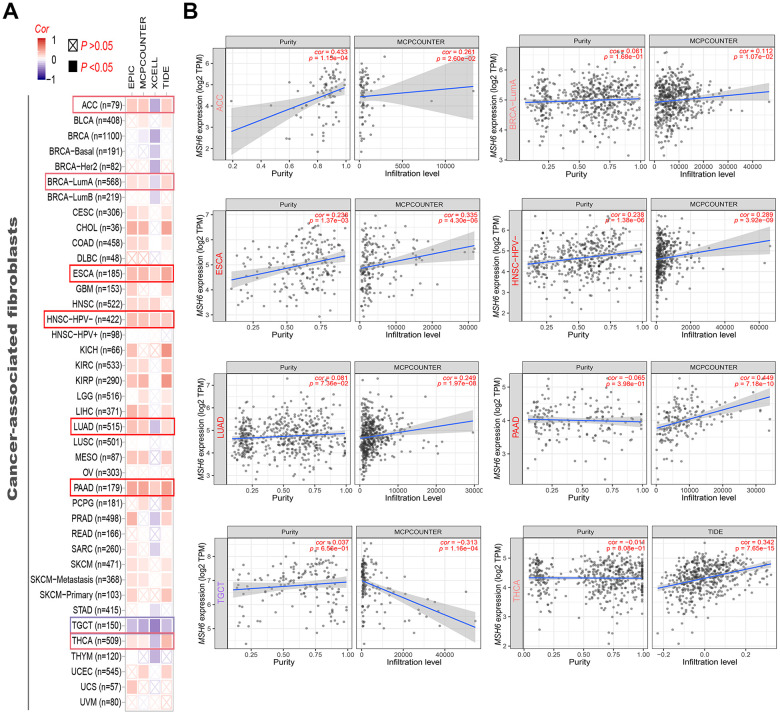
**Correlation analysis between MSH6 gene expression and immune infiltration of cancer-associated fibroblasts.** (**A**) Different algorithms (including MCPCOUNTER, EPIC, XCELL and TIDE) were applied to evaluate the relationship between MSH6 expression and the immune infiltration level of cancer-associated fibroblasts for all TCGA tumors. (**B**) The scatterplot data of the selected tumor generated using one of the algorithms were supplied.

Figure 5 shows the cancer-associated fibroblast infiltration in different cancer types of TCGA and potential relationships between MSH6 gene expression. We found that the cancer-associated fibroblasts’ estimated infiltration value for the TCGA tumors of ESCA, HNSC (HPV -) and PAAD analyzed based on all algorithms was statistically positively related with the expression of MSH6, and only negatively related in TGCT tumors. Moreover, we also noted that the estimated infiltration value of CD8^+^ T-cells immune infiltration analyzed based on all or most of the algorithms was statistically positively related with the MSH6 expression in PAAD and THYM tumors, but was negatively correlated in UCEC and TGCT tumors (Supplementary Figure 3). In Figure 5B and Supplementary Figure 3B, there are scatterplot data of the above-mentioned tumor generated using one of the algorithms. For example, the expression of MSH6 gene in THCA was positively related with the cancer-associated fibroblasts’ infiltration level (cor=0.342, P=7.65e-15) based on the TIDE algorithm (Figure 5B). The above results indicate that tumor infiltrating immune cells are important components of the tumor microenvironment and were closely related to the occurrence, development, or metastasis of cancer.

### Function enrichment analysis of MSH6-related genes

**Figure 6:**
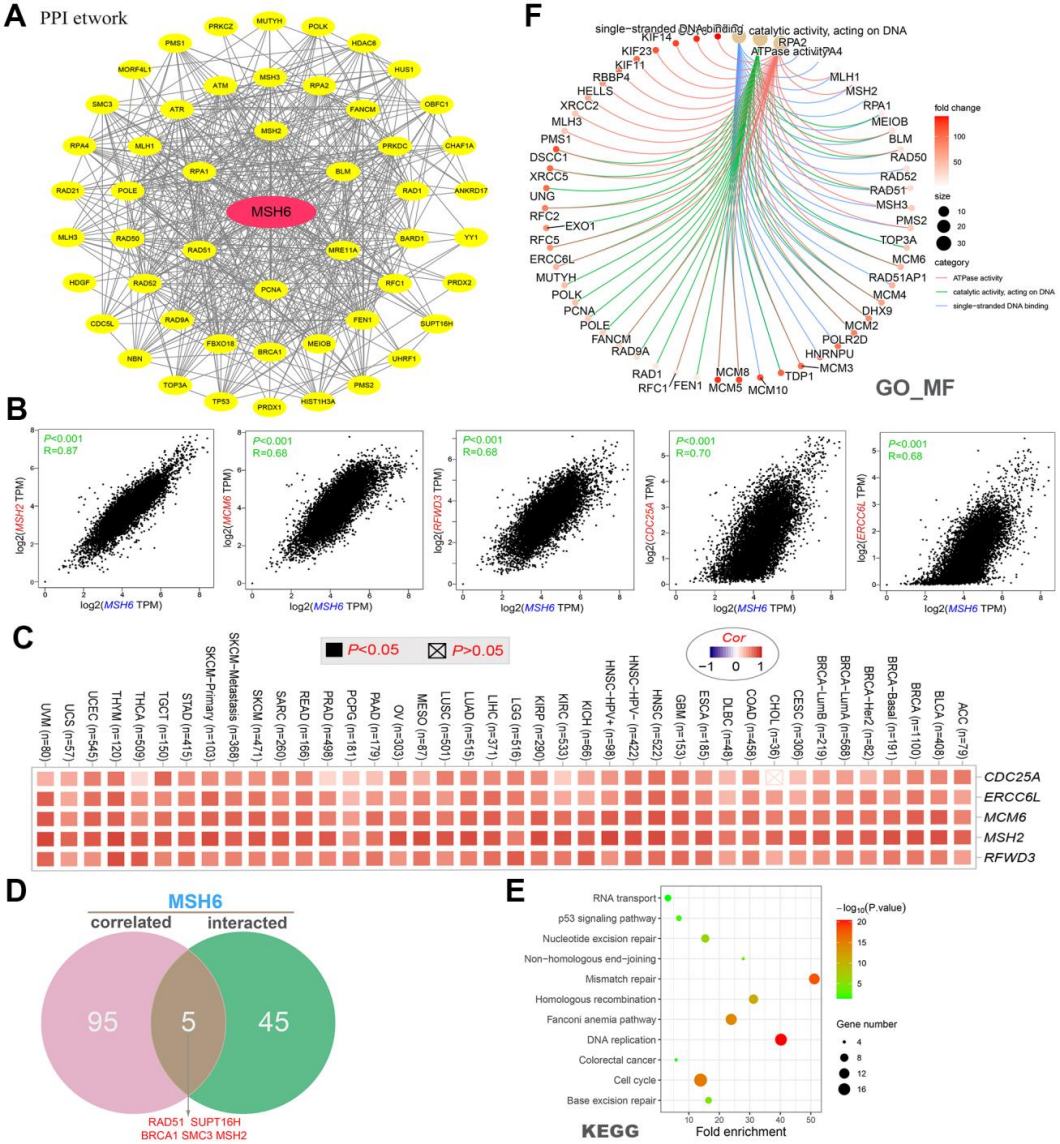
**MSH6-related gene function enrichment analysis.** (**A**) On the basis of the STRING tool, a total of 50 available experimentally verified MSH6-binding proteins were obtained. (**B**) The top 100 MSH6-correlated genes in the TCGA project were gained by using the GEPIA2 tool, and it analyzed the expression correlation between MSH6 and the top 5 targeting genes (including MSH2, MCM6, RFWD3, CDC25A and ERCC6L). (**C**) It displayed the corresponding heatmap data of the selected targeting genes in the TCGA detailed cancer type are displayed. (**D**) Intersection analysis of MSH6-correlated genes and MSH6-binding protein. (**E**) KEGG pathway analysis on the basis of MSH6-correlated genes and MSH6-binding protein. (**F**) The cnetplot for GO enrichment analysis (molecular function data) based on MSH6-correlated genes and MSH6-binding protein.

On the basis of the STRING tool, a total of 50 available experimentally verified MSH6-binding proteins were obtained. Figure 6A shows the interaction network of these proteins. Moreover, the GEPIA2 tool was applied to combine the dataset of all tumors and adjacent normal tissues of TCGA to gain the top 100 targeted genes correlated to the expression of MSH6. In Figure 6B, the MSH6’s expression level was positively correlated with that of MCM6 (Mini-chromosome maintenance complex component 6) (R=0.68), MSH2 (R=0.87), CDC25A (Cell division cycle 25A) (R=0.70), RFWD3 (Ring finger and wd repeat domain 3) (R=0.68)and ERCC6L (ERCC excision repair 6 like and spindle assembly checkpoint helicase) (R=0.68) genes (all P <0.001). We obtained the heatmap data by using the TIMER2 online tool, which further indicated that the expression level of MSH6 and these five genes were positively related to the most tumor types of TCGA (Figure 6C). In Figure 6D, Jvenn tool was used for intersection analysis of the above two groups to obtain 5 common members, including RAD51 (RAD51 recombinase), SUPT16H (SPT16 homolog, facilitates chromatin remodeling subunit), BRCA1 (BRCA1 DNA repair associated), SMC3 (Structural maintenance of chromosomes 3) and MSH2. Similarly, both scatterplot data and heatmap data indicated that the MSH6 expression level was positively related to that of RAD51(R=0.65), BRCA1 (R=0.57), SMC3 (R=0.58) and SUPT16H (R=0.57) genes (all P <0.001), and it was also true in most of TCGA tumor types (Supplementary Figure 4A, 4B).

Moreover, the two datasets were combined to perform GO and KEGG pathway enrichment analysis. In Figure 6E, the enrichment analysis of the KEGG pathway demonstrates that “DNA replication”, “mismatch repair” and “cell cycle” may be included in the effect of MSH6 in tumorigenesis and development. GO enrichment analysis data will illustrate that majority of these genes are related to the pathways or cellular biology of DNA metabolism, including ATPase activity, DNA recombination, catalytic activity acting on DNA, double−strand break repair, chromosomal region, nuclear chromosome, etc. (Figure 6F and Supplementary Figure 4C, 4D).

## DISCUSSION

As one of the three most important mismatch repair genes in the MutS family, MSH6 has been shown to be involved in the occurrence and development of many different cancers, including colorectal cancer, endometrial cancer, prostate cancer, pituitary adenoma and osteosarcoma [10–13, 17]. Edelmann’s results revealed that mutations in the MSH6 gene increased cancer susceptibility and may be directly related to hereditary cancer predisposition syndrome and certain sporadic tumors without microsatellite instability [20]. Nevertheless, it remains unclear whether MSH6 plays a role in the pathogenesis of different tumors through some common molecular mechanisms. To address this, we performed pan-cancer analysis of MSH6 gene across 33 different cancer types, based on the data of TCGA, CPTAC, and GEO databases, as well as the molecular characteristics of gene expression, genetic alteration, or protein phosphorylation. In this study, we provided new insights into the underlying molecular mechanisms of MSH6 in the pathogenesis or clinical prognosis of different cancers.

MSH6 mRNA was highly expressed in most tumors of TCGA and corresponded to both the increased MSH6 protein expression and target gene expression of corresponding tumors, indicating that MSH6 has functional activity in these tumors. Wilczak et al. [12] performed immunohistochemical analysis on a tissue microarray of 11152 prostate cancer specimens and showed that MSH6 overexpression is common in prostate cancer and is associated with poor survival prognosis and genetic instability. Similarly, our previous study on the expression level of MSH6 in osteosarcoma tissues found that MSH6 was significantly overexpressed in the pathological tissues of osteosarcoma, and silencing MSH6 gene may have a better effect on inhibiting osteosarcoma cell proliferation and promoting cell apoptosis [17].

In this work, GEPIA2 tool was used to detect the correlation between the expression of MSH6 and the prognosis of different tumors in TCGA. We found that high expression of MSH6 was linked to poor prognosis of OS for cancers of ACC, BLCA, KIRP, LGG, and SARC, while the opposite was true for KIRC and THYM. A number of recent studies have also shown that the expression of MSH6 was associated with the poor survival of cancer patients such as LGG and SARC, and that MSH6 gene mutations may increase the risk of certain tumors with lower prevalence, such as ACC [21–23]. Nevertheless, However, no study has been reported on the clinical prognosis of MSH6 expression in BLCA, KIRP, KIRC and THYM tumors. Although the survival prognosis analysis data of MSH6 gene showed different conclusions in different tumors, we believe that the abnormal expression of MSH6 gene is closely related to the poor survival prognosis of most tumors.

A number of recent studies have reported that MSH6 gene expression is associated with an increased risk of breast or ovarian cancer [11, 24, 25]. Nevertheless, we failed to observe the correlation between MSH6 expression and the survival prognosis of patients with breast or ovarian cancer in the TCGA-BRCA/OV cohort. Different data processing or updated survival information may contribute to this result. Consequently, based on the survival data of the Kaplan-Meier plotter with Affymetrix 202911_at and 211449_at microarrays [26], we observed that the high expression of MSH6 was associated with poor prognosis of OS, RFS, PFS and DMFS prognosis in breast cancer cases. Furthermore, we also observed a correlation between MSH6 high expression and poor prognosis of OS, PFS and PPS in ovarian cancer cases. Similar to previous studies, our research also indicated that MSH6 may be a susceptibility gene for breast cancer or ovarian cancer, and the expression of MSH6 gene may cause poor survival prognosis in these two cancer patients. Nevertheless, we believe that more in-depth molecular experimental evidence is still needed to confirm whether the high expression of MSH6 plays an essential role in the above mentioned tumor initiation process, or is just the result of normal tissues resisting tumor progress.

Using the CPTAC dataset, we first explored the molecular mechanism of MSH6 protein in breast cancer, colon cancer, lung adenocarcinoma, clear cell renal cell carcinoma, ovarian cancer, and uterine corpus endometrial carcinoma from the perspective of total protein and phosphoprotein. The results of this study demonstrated that, compared with normal tissues, total MSH6 protein was highly expressed and phosphorylated at S830 and S227 sites in MutS_III domain in primary tumor tissues. Although the S227 and S830 sites of MSH6 phosphorylation in the cell cycle have been experimentally confirmed [18], there is no research on the potential role of MSH6 phosphorylation at S227 and S830 sites during cell cycle regulation. Hence, this may require additional molecular experiments to further evaluate the potential role of MSH6 phosphorylation at S227 and S830 sites in the initiation and progression of different tumors.

Cancer-associated fibroblasts in the stroma of the tumor microenvironment were reported to be involved in regulating the functions of various tumor-infiltrating immune cells [27, 28]. Hence, to clarify the relationship between MSH6 expression and tumor-infiltrating immune cells, we investigated the relationship between MSH6 expression and cancer-associated fibroblasts, as well as CD8^+^ T-cells immune infiltration levels of different cancer types. Interestingly, MSH6 expression was positively correlated with cancer-associated fibroblasts and CD8^+^ T-cells infiltration levels in most cancer types, including PAAD, ESCA, HNSC (HPV -) and THYM, etc. Not surprisingly, this may be related to microsatellite instability (MSI). MSI refers to changes in microsatellite length caused by insertion or deletion of repeating units in tumors, which is mainly related to germline mutations in genes such as MLH1, PMS2, MSH2, and MSH6 [29]. Increasingly, MSI has been shown to be associated with a large number of tumor-infiltrating lymphocytes, which provides indirect evidence for the special role of the antitumoral immune response in such tumors, possibly due to the increased neoantigen production [30–32].

In this study, we determined the potential role of “DNA replication”, “mismatch repair”, “cell cycle”, “ATPase activity” and DNA metabolism in the etiology or pathogenesis of cancer through a series of enrichment analyses on MSH6-binding protein and MSH6 expression-related genes across all tumors. As a mismatch binding factor, MSH6 can repair mismatched bases in DNA replication, gene damage, and recombination to maintain the stability of genetic information [33–35]. A large number of studies have confirmed that MSH6 can promote tumor genesis and development through the interaction with histone H3Kme36, chromatin complex effects, and genomic microsatellite instability and other mechanisms [29, 36, 37]. These findings may help to understand the potential role of the MSH6 gene in the pathogenesis of different tumors.

Altogether, our first pan-cancer analysis of MSH6 showed that MSH6 is expressed in most cancers, and the MSH6 expression is significantly correlated to the clinical prognosis of cancer patients, protein phosphorylation, and immune cell infiltration. These data provided a relatively comprehensive understanding of the oncogenic effects of MSH6 across different tumors, which helps us to know the effect of MSH6 in tumorigenesis in the view of clinical tumor samples.

## MATERIALS AND METHODS

### Expression analysis of gene

The present study shows that we first entered “MSH6” in the “Gene_DE” module of Tumor Immune Estimation Resource (TIMER2, http://timer.cistrome.org/) and found the differences of MSH6 expression between adjacent normal tissues and thirty-three different tumors or specific tumor subtype tissues in the TCGA project (Supplementary Table 1). Some tumors, however, that have no normal tissue or a high degree of normal tissue deficiency in the TCGA project, such as TCGA-DLBC (Lymphoid Neoplasm Diffuse Large B-cell Lymphoma), TCGA-LGG (Brain Lower Grade Glioma), etc., the corresponding normal tissues in the Genotype-Tissue Expression (GTEx) database were obtained and it applied the Gene Expression Profiling Interactive Analysis’ “Expression analysis-Box Plots” module (GEPIA2, http://gepia2.cancer-pku.cn/#analysis) [38] to find differential expression between these tumor tissues and the corresponding normal tissues (setting: *P*-value cutoff = 0.01, log_2_FC (fold change) cutoff = 1, and “Match TCGA normal and GTEx data”). The UALCAN portal (http://ualcan.path.uab.edu/analysis-prot.html) allowed us to conduct the analysis in protein expression level on the dataset of CPTAC (Clinical proteomic tumor analysis consortium) [39]. Hence, we input “MSH6” in the UALCAN portal’s “CPTAC Analysis” module to seek the total protein or phosphoprotein expression levels of MSH6 (NP_000170.1) between the TCGA project’s primary tumor and normal tissues. Herein, six available datasets for tumors have been selected, namely, colon cancer, breast cancer, ovarian cancer, LUAD (Lung adenocarcinoma), clear cell RCC (Renal cell carcinoma), and UCEC (Uterine corpus endometrial carcinoma). Finally, through the GEPIA2 “Pathological Stage Plot” module, the MSH6 expression violin plots in various pathological stages (stage I, II, III, IV and V) of the tumors in TCGA were all obtained. The log2 [Transcripts per million (TPM) + 1] converted expression data were used to violin plots or box.

### Survival analysis

In order to understand the effect of MSH6 gene expression on the survival and prognosis of all TCGA tumors. The current study shows that the GEPIA2 “Survival Map” module [38] was applied to evaluate the relationship between MSH6 gene expression and overall survival (OS) and Disease-free survival (DFS) of all TCGA tumors (settings: cutoff-high value: 50%, cutoff-low value: 50%). Log-rank tests were used as the hypothesis tests. Moreover, we also applied the GEPIA2 “Survival Analysis” module to gain survival plots with MSH6 expression significance correlated in all TCGA tumors.

### Genetic alteration analysis

The Cancer Genomics cBioPortal portal (https://www.cbioportal.org/) [40] provides a network resource to explore, visualize, and analyze multidimensional cancer genomic data, which allows us to interactively explore the genetic changes across genes, samples, and pathways, and link these to clinical outcomes when available in the underlying data. In this study, we selected “TCGA Pan Cancer Atlas Study” in “Quick Select” section of the cBioPortal web and entered into “MSH6”to find the genetic alteration characteristics of MSH6. Next, we observed the alteration frequency results, structural variants, mutation type, and CNA (Copy number alteration) of all TCGA tumors within the “Cancer Types Summary” module. Finally, the “comparison/survival” module was applied to analyze the disease-free, progression-free and overall survival differences of UCEC cancer patients with or without MSH6 genetic alteration. And Kaplan-Meier survival curves were used for data visualization.

### Immune infiltration analysis

When logging into the TIMER2 web server, we chose the “Immune-Gene” module and entered “MSH6” to find the association between MSH6 expression and immune infiltrates for all the tumors in the TCGA project. In this study, we selected immune cells, including CD8^+^ T-cells and cancer-associated fibroblasts. Next, the potential relations between the immune infiltration level of different immune cells and the MSH6 expression for all TCGA tumors was estimated by the TIMER, XCELL, CIBERSORT-ABS, MCPCOUNTER, CIBERSORT, EPIC, QUANTISEQ, and TIDE algorithms. Additionally, the *P*-values were gained by the rank correlation test of Spearman after purity adjustment, and a heatmap and a scatter plot were visualized to the final results.

### MSH6-related gene enrichment analysis

We used the single protein name (“MSH6”) firstly and an organism (“Homo sapiens”) to screen available experimentally verified MSH6-binding proteins in the “Protein By Name” module of STRING website (https://string-db.org/). At the same time, the following main parameters were set: minimum required interaction score [“Low confidence (0.150)”], active interaction sources (“experiments”), meaning of network edges (“evidence”) and max number of interactors to indicate (in the 1st shell, there is “no more than 50 interactors”). Finally, we gained 50 available experimentally verified MSH6-binding proteins and constructed a network of these protein interactions. Through Cytoscape software, the PPI network was visualized.

On the basis of the dataset of all tumors and adjacent normal tissues of TCGA project, we gained the top 100 targeted genes linked to the MSH6 expression by using GEPIA2’s “Similar Gene Detection” module. Then, pairwise gene Pearson correlation analysis was performed to analyze the correlation between MSH6 and the top 5 targeting genes by using the “Correlation Analysis” module of GEPIA2. *P*-values and the correlation coefficient (R) were displayed in the plot. Finally, TIMER2 “Gene_Corr” module was applied to create the selected genes’ heatmap data. The heatmap indicated the *P*-values in the Spearman’s rank correlation test and partial correlation (cor) values after purity adjustment.

Jvenn (http://bioinformatics.psb.ugent.be/webtools/Venn/) [41] is a new JavaScript library that helps us process lists and generate Venn diagrams by classical or Edwards-Venn layouts, thereby enhancing its readability function. In this paper, we applied the Jvenn online tool to implement an intersection analysis, of selected genes to obtain genes related to and interacting with MSH6 expression. Subsequently, to further understand the functions of these genes, the two sets of data were combined to conduct GO (Gene ontology) and KEGG (Kyoto Encyclopedia of Genes and Genomes) path analysis. Specifically, we first use the DAVID online tool (visualization, database for annotation and integrated discovery, https://david.ncifcrf.gov) to obtain the functional annotation chart data. Next, the “cairo”, “stringr” and “ggplot2” R packages were applied to visualize these genes’ enrichment pathways, and the “clusterProfiler” R package was applied to perform the GO (Gene Ontology) enrichment analysis. Finally, by using CNET plot function, the data of CC (Cellular component), BP (Biological process), and MF (Molecular function) are turned to cnetplot. This paper applied the R language software [R-4.1.0, 64-bit] (https://www.r-project.org/). Two-tailed P <0.05 was statistically significant.

### Data availability statement

In the study, the original contributions presented are included in the article/supplementary materials, and further inquiries can be directed to the corresponding author.

## Supplementary Material

Supplementary Figures

Supplementary Table 1
