# Supplementary material for: An integrative pan-cancer analysis reveals the oncogenic role of mutS homolog 6 (MSH6) in human tumors
Source: Aging (Albany NY). 2021 Dec 7;13(23):25271–90. doi: 10.18632/aging.203745 (PMC8714153; doi:10.18632/aging.203745)
Supplement: Supplementary Figures [file aging-13-203745-s001.pdf]

SUPPLEMENTARY FIGURES

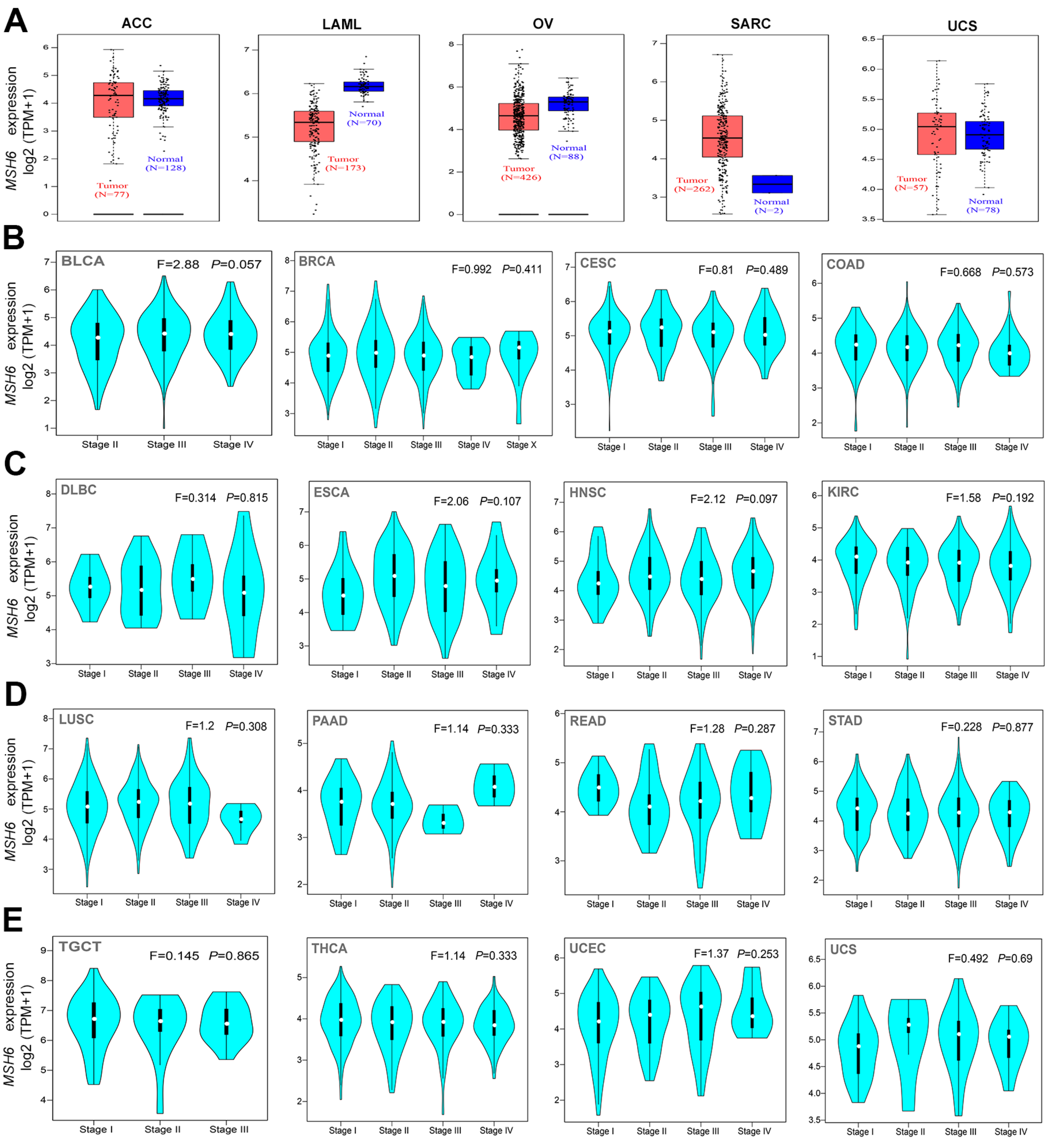

Supplementary Figure 1. Expression level of MSH6 gene in different tumors and pathological stages. (**A**) The corresponding normal tissues in the GTEx database were used as controls, and GEPIA2 was used to analyze the expression status of MSH6 gene in ACC, LAML, OV, SARC and UCS tumors. Expression levels of the MSH6 gene by different pathological stages of BLCA, BRCA, CESC, COAD (**B**); DLBC, ESCA, HNSC, KIRC (**C**); LUSC, PAAD, READ, STAD (**D**); and TGCT, THCA, UCEC, UCS (**E**).

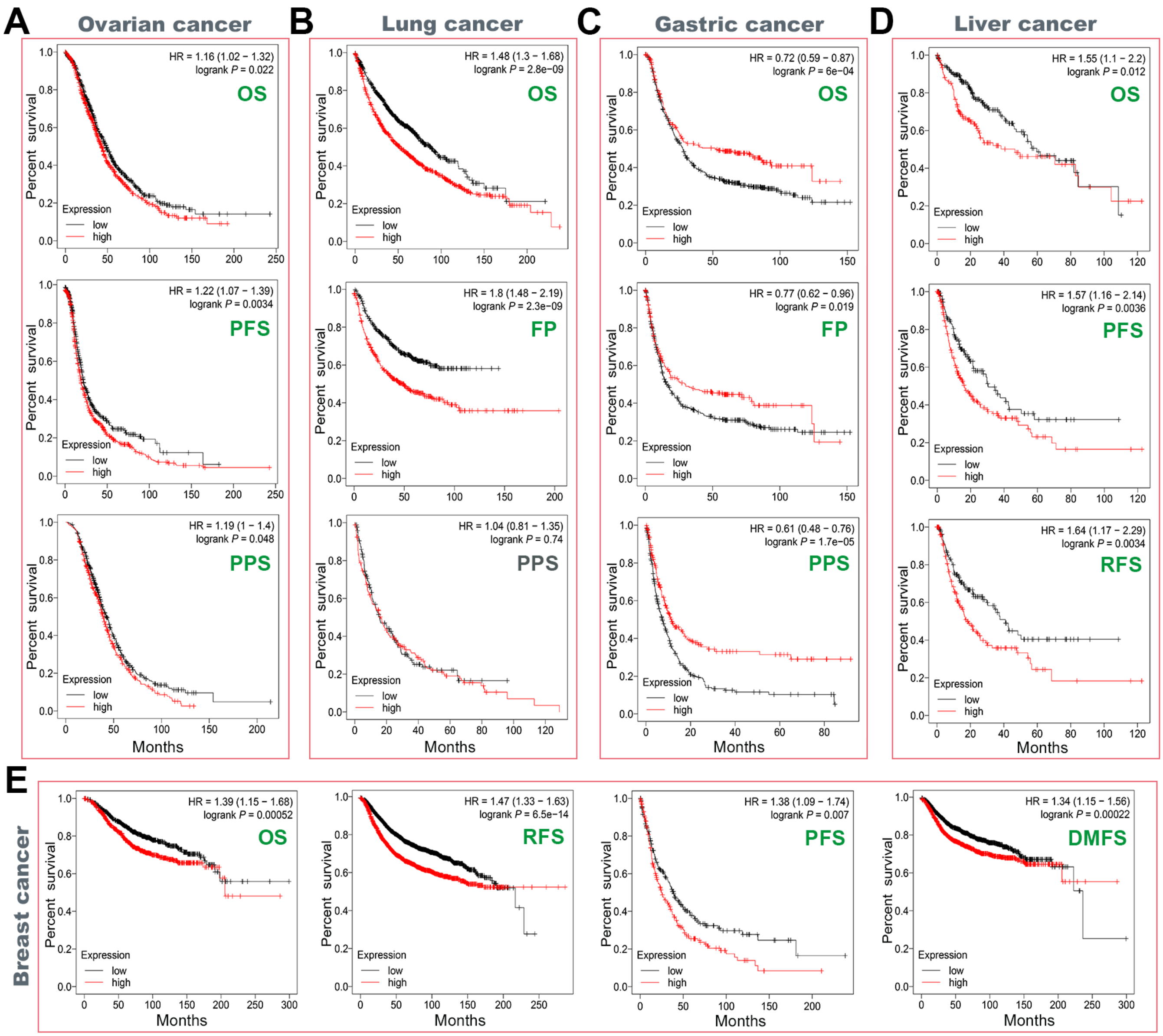

Supplementary Figure 2. Kaplan-Meier plotter was used to analyze the correlation between MSH6 gene expression and cancer prognosis. Based on the expression level of MSH6 gene in ovarian cancer (**A**), lung cancer (**B**), gastric cancer (**C**), liver cancer (**D**) and breast cancer (**E**) cases, we performed a series of survival analysis using Kaplan-Meier plotter, including OS, DMFS, RFS, PFS, PPS, and FP.

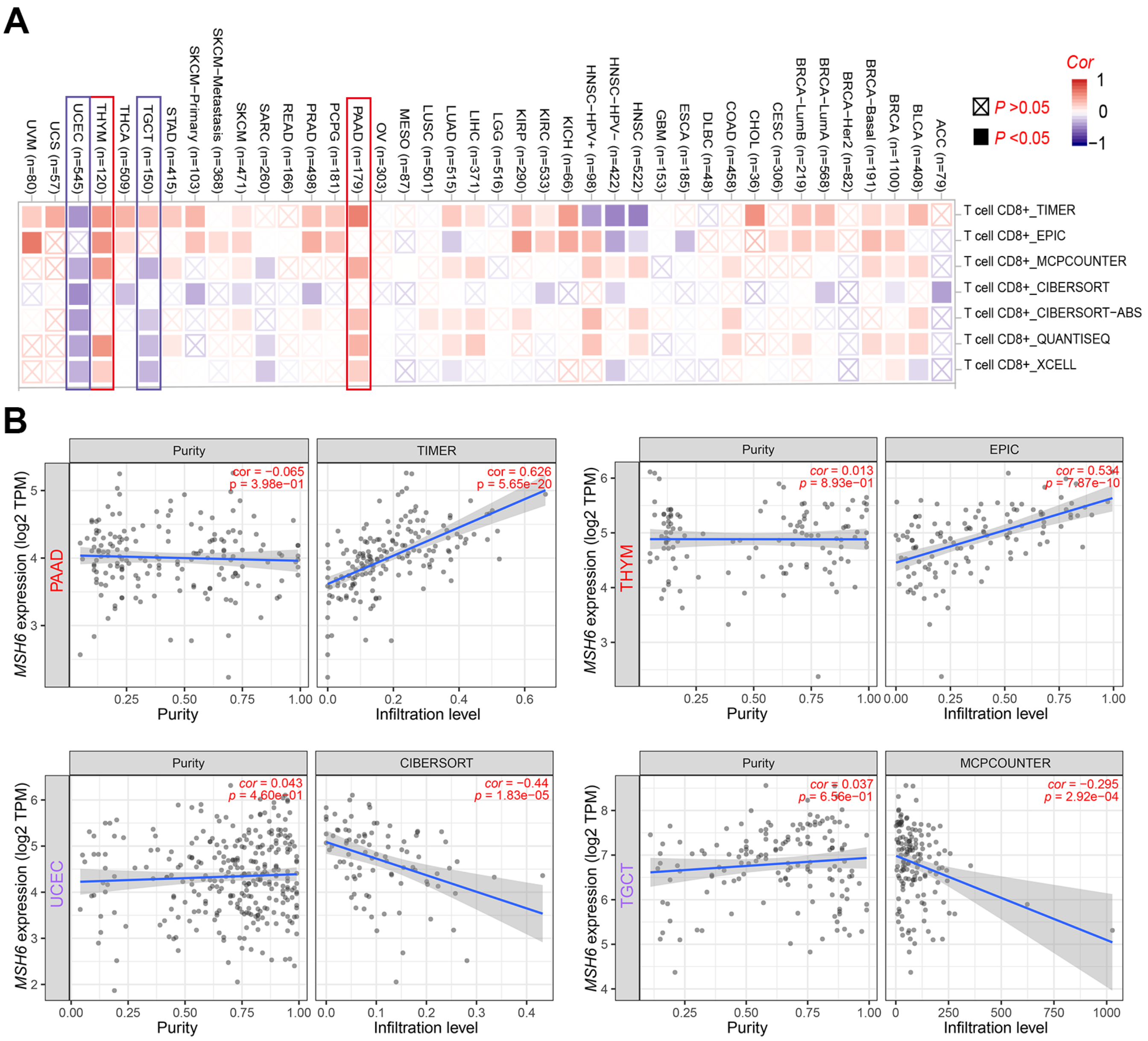

Supplementary Figure 3. Correlation analysis between MSH6 gene expression and immune infiltration of CD8+ T-cells. (**A**) Different algorithms (including TIMER, EPIC, MCPCOUNTER, CIBERSORT, CIBERSORT-ABS, QUANTISEQ and XCELL) were applied to evaluate the relationship between MSH6 expression and the immune infiltration level of CD8+ T-cells for all TCGA tumors. (**B**) The scatterplot data of the selected tumor generated using one of the algorithms were supplied.

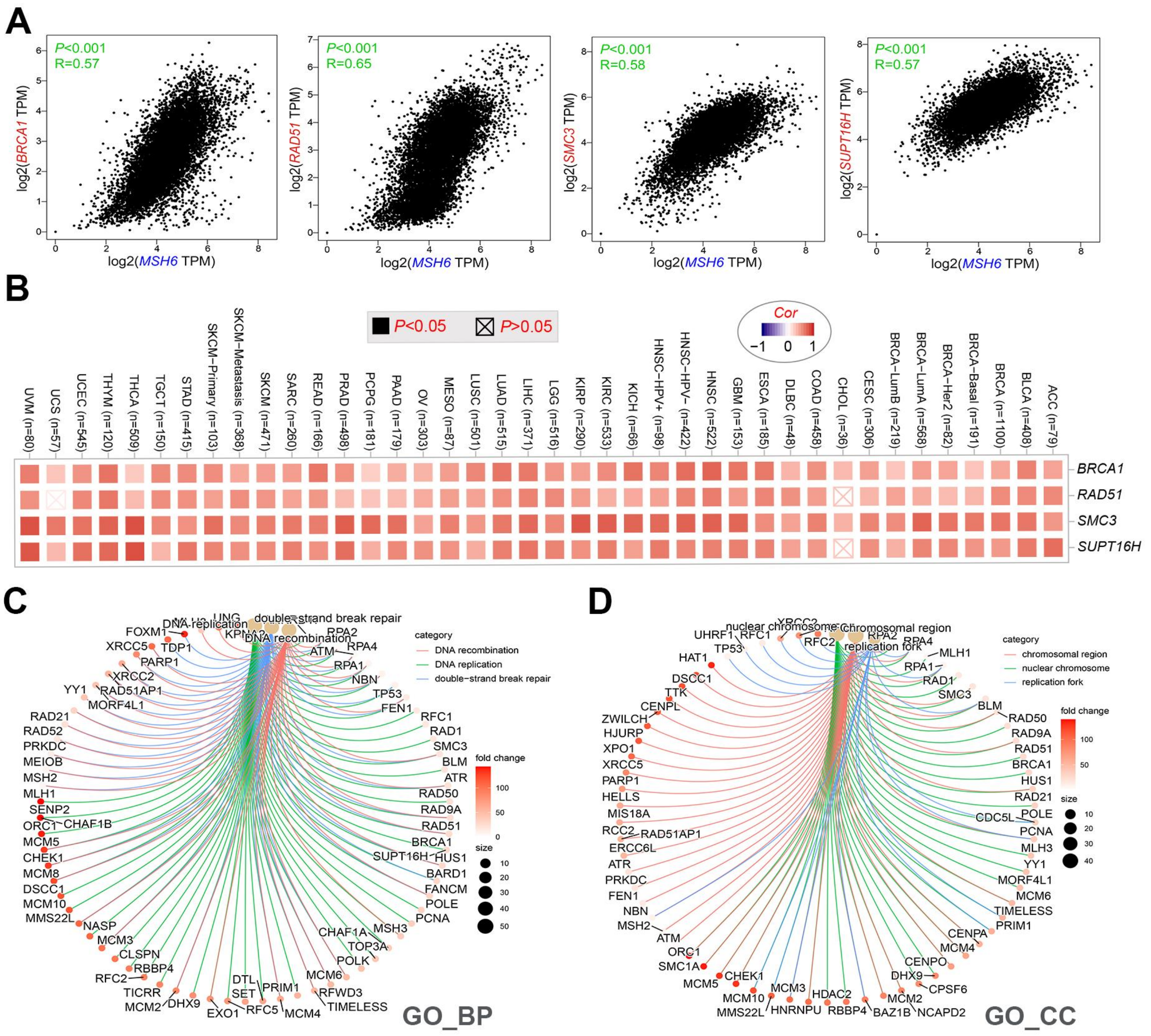

Supplementary Figure 4. GO-biological process/cellular component analysis of MSH6-related genes. (**A**) Using the GEPIA2 approach, we analyzed the expression correlation between MSH6 gene and crossover genes (including BRCA1, RAD51, SMC3 and SUPT16H). (**B**) The corresponding heatmap data of the crossover genes in the TCGA detailed cancer type are displayed. (**C**) The cnetplot for the biological process data in GO enrichment analysis based on MSH6-correlated genes and MSH6-binding protein. (**D**) The cnetplot for the cellular component data in GO enrichment analysis based on MSH6-correlated genes and MSH6-binding protein.
